# Identifying patients with cognitive impairment in a large health system: an innovative initiative to facilitate detection in primary care

**DOI:** 10.1002/alz.084535

**Published:** 2025-01-09

**Authors:** Barak Gaster, Monica Zigman Suchsland, Annette L. Fitzpatrick, Joshua M. Liao, Basia Belza, Amy P Hsu, Sarah McKiddy, Benjamin S. Olivari, Jaqueline G. Raetz

**Affiliations:** ^1^ University of Washington, Seattle, WA USA; ^2^ University of Texas Southwestern, Dallas, TX USA; ^3^ Centers for Disease Control and Prevention, Atlanta, GA USA

## Abstract

The prevalence of Alzheimer’s disease and related disorders (ADRD) is rising, and primary care providers (PCPs) will increasingly play a role in its detection. We developed an evidence‐based intervention to facilitate cognitive evaluation in primary care. We then implemented it as a set of web‐based trainings which were combined with specific tools embedded in the electronic health record (EHR) for PCPs to use in their practices. The model was adapted from the Gerontological Society of America’s KAER (Kickstart, Assess, Evaluate, Refer) Toolkit, and rolled out across 14 community‐based primary care clinics in a large health system. Outcome measures included: 1) the number of cognitive assessments entered into the EHR; 2) the number of patients diagnosed with mild cognitive impairment (MCI); and 3) change in PCP confidence and attitudes in identifying and managing dementia assessed by a validated survey tool, the General Practitioner’s Attitudes and Confidence Scale for Dementia (GPACS‐D). The number of cognitive assessments entered into the EHR in the six months before the intervention compared to the six months after increased from a mean of 5.2 to 21.2 per month (p<0.01); the number of patients newly diagnosed with MCI increased from a mean of 23.5 to 32.2 per month (p = 0.03); and fifty PCPs who completed the GPACS‐D survey reported significant improvement in confidence and attitudes (mean total attitude and confidence score increase from 3.33 to 4.09, p<0.01). This data suggests an initiative like this can improve cognitive evaluation and increase identification of MCI across a large primary care network. The initiative’s methods are designed to be easy to replicate, without requiring hiring new staff or building new services, making it highly scalable. The educational videos and EHR tools developed are available at no charge to anyone who wants to adapt this approach. Easy to deploy, with clear, reproducible outcomes, it has the potential to improve ADRD care. As new therapeutics for ADRD become available, initiatives such as this will increasingly be needed to improve referrals to specialists, foster an engaged primary care workforce for collaboration, and improve the lives of patients living with ADRD.

